# Hematological Side Effects of Immune Checkpoint Inhibitors: The Example of Immune-Related Thrombocytopenia

**DOI:** 10.3389/fphar.2019.00454

**Published:** 2019-04-26

**Authors:** Roser Calvo

**Affiliations:** Patient Safety, Safety Science, AstraZeneca Pharmaceuticals, Gaithersburg, MD, United States

**Keywords:** immune-related thrombocytopenia, immune-related adverse events, ir-AEs, immune checkpoint inhibitors, immune-related hematological adverse events, immune thrombocytopenia

## Abstract

Immune-related hematological adverse events are amongst the rare but potentially life-threatening complications of immune checkpoint inhibitors. The spectrum of these toxicities is broadening as the number of patients exposed to these agents is increasing. Yet, they are still relatively unknown to many clinicians, possibly due to a lack of specific diagnostic criteria, which poses a challenge for their recognition and proper reporting, and partly due to their low incidence, often too low to be noted in most clinical trial publications. Since early detection and prompt intervention are crucial to prevent fatal consequences, it is of outmost importance that medical staff and patients be aware of these potential toxicities and learn to recognize and treat them adequately. This publication outlines strategies and offers guidance on the detection, diagnosis, risk assessment, monitoring and management of immune-related thrombocytopenia, a relatively common example of immune-related hematological toxicity of immune checkpoint inhibitors.

## Introduction

Immune checkpoints inhibitors (ICIs) targeting PD-1/PD-L1 (programmed cell death receptor-1 or ligand-1) and CTLA-4 (lymphocyte-associated protein 4) have been associated with a growing list of autoimmune-like safety complications known as immune-related adverse events (ir-AEs), which can affect virtually any organ, mainly skin, gastrointestinal, hepatic, pulmonary, mucocutaneous, endocrine, and less frequently others including the hematological system.

With the increasing number of approved ICIs, new indications, and number of patients exposed to them, the repertoire of hematological ir-AEs (hem-irAEs) now extends to entities as varied as pure red cell aplasia (Gordon et al., 2009; Nair et al., 2016; Yuki et al., 2017), aplastic anemia/bone marrow failure (Comito et al., 2017; Michot et al., 2017; Helgadottir et al., 2017; Meyers et al., 2018), hemophilia A (Delyon et al., 2011; Lozier, 2012), acute thrombosis (Kunimasa et al., 2018), large granular lymphocytosis (Wei et al., 2012), hemophagocytic lymphohistiocytosis (Sadaat and Jang, 2018), macrophage activation syndrome (Malissen et al., 2017), eosinophilia (Bernard-Tessier et al., 2017), and hematological cytopenias affecting one or more hematological cell lines. Literature reports include cases of ir-neutropenia (Akhtari et al., 2009; Wei et al., 2012; Simeone et al., 2014; Wozniak et al., 2015; Sun et al., 2018), autoimmune hemolytic anemia (Kong et al., 2016;Nair et al., 2016; Palla et al., 2016; Schwab et al., 2016; Cooling et al., 2017; Khan et al., 2017; Tardy et al., 2017; Sun et al., 2018), ir-thrombocytopenia (ir-TCP) (Chung et al., 2010; Ahmad et al., 2012; Hilmi Atay et al., 2015; Kopecky et al., 2015; Solomon, 2015; Bagley et al., 2016; Inadomi et al., 2016; Kanameishi et al., 2016; Karakas et al., 2017; Le Burel et al., 2017; Pföhler et al., 2017; Shiuan et al., 2017; Jotatsu et al., 2018; Sun et al., 2018), and pancytopenia (Ku et al., 2010; Di Giacomo et al., 2011; du Rusquec et al., 2014). Although hem-irAEs are rare, with ir-cytopenias reported with PD-1/PD-L1 inhibitors at a frequency of 0.5% for CTCAE (Common Terminology Criteria for Adverse Events) grade ≥2 events (Delanoy et al., 2019), they can be life-threatening and warrant early recognition and appropriate patient management to prevent potentially fatal outcomes. This review focuses specifically on ir-TCP as the most common type of hem-irAEs along with autoimmune hemolytic anemia and neutropenia, each occurring in 26% of patients with a reported hem-irAE during PD-1/PD-L1 treatment registered in three French pharmacovigilance databases (Delanoy et al., 2019). Moreover, compared with TCP of conventional anticancer drugs, clinicians are less familiar with ir-TCP, which may lead to misdiagnosis of an entity that is clinically serious and for which delaying adequate care could lead to a worse prognosis. Despite the noted limitations due to the rarity of ir-TCP and consequently the retrospective nature of most series from which data for this publication is extracted, we hope this review will increase the physician’s familiarity with clinical aspects of ir-TCP and algorithms for optimal management and minimization of this toxicity.

Mechanistically, ir-AEs are thought to be caused by a reinvigoration of exhausted T-cells once the ICI exerts the desired effect on the PD-1/PD-L1 or CTLA-4 pathway, evoking inflammation and ultimately leading to the occurrence of ir-AEs. Other immune cells may play a role, including B cells that produce antibodies that may mediate the toxicity. Although the precise pathogenesis of ir-TCP is unclear, the reaction is thought to be triggered by ICI-induced antiplatelet antibody production via an autoimmunity activation, which is supported by high levels of platelet-associated autoantibodies in many patients with ir-TCP (Pföhler et al., 2017; Jotatsu et al., 2018; Leroy et al., 2018).

While TCP of unspecified etiology is relatively frequent with ICIs (Ansell et al., 2015), ir-TCP is reported at an incidence of around 1–2% (Chung et al., 2010; Friedman et al., 2016; Kourie et al., 2016; Shiuan et al., 2017; Le Burel et al., 2017; Sun et al., 2018). Because there may be alternative explanations for the TCP, there is a reasonable risk that ir-TCP may be under-recognized and treatment delayed, with an inherent risk of potentially fatal bleeding complications. There is increasing need for guidance to aid clinicians and investigators to anticipate, recognize, mitigate, monitor, and manage suspected cases of ir-TCP during ICI therapy. This paper offers a review of the current available reports in the literature (Chung et al., 2010; Ahmad et al., 2012; Hilmi Atay et al., 2015; Kopecky et al., 2015; Solomon, 2015; Bagley et al., 2016; Inadomi et al., 2016; Kanameishi et al., 2016; Karakas et al., 2017; Le Burel et al., 2017; Pföhler et al., 2017; Shiuan et al., 2017; Jotatsu et al., 2018).

## How Should We Define and Diagnose Immune-Related Thrombocytopenia?

A practical definition of ir-TCP would enhance this AE recognition, optimal patient management, and case reporting. The challenge is that ir-TCP can mimic virtually any other type of TCP, with no specific biomarkers or diagnostic tests, making it a diagnosis of exclusion after ruling out other more common causes of TCP. A broad definition (e.g., “TCP following treatment with an ICI that is not attributable to another cause and/or has a clinical phenotype that suggests an immune event”) may be helpful if supported by certain clinical factors that point to an underlying autoimmunity, such as those provided in Table 1. At a practical level, the possibility of an underling ir-TCP should always be considered with any new unexplained decrease in platelet counts, particularly when work-up does not uncover other common causes of TCP. Consistent with other irAEs, ir-TCP develops generally within the first 12 weeks of ICI initiation (Shiuan et al., 2017; Delanoy et al., 2019) but onset can occur at any time: at the beginning, a few months into treatment, and after immunotherapy termination. Delayed onset cases have been reported as late as two months after the cessation of therapy (Hilmi Atay et al., 2015; Leroy et al., 2018).

**Table 1:** Diagnostic criteria suggestive of immune-related thrombocytopenia.

**(1) Absence of any other factors likely to cause TCP**	
**Non-immune**	**Immune**
Prior or concomitant chemo-radiotherapy. Bone marrow tumor infiltration. Hematological malignancies (MDS, MPD). Platelet sequestration (hypersplenism, liver diseases) Platelet consumption (TMA, TTP, DIC)	Primary immune TCP (ITP). Other causes of secondary immune TCP: infections (HIV, HCV, CMV, EBV, Parvovirus, *H. Pylori*, vaccinations with live attenuated virus), lymphoproliferative disorders (CLL, HL, NHL, LGL), autoimmune conditions (thyroid disease, SLE, APS), other drug-induced TCP (heparin/HIT, herbal remedies, over the counter products, drugs other than the ICI), post-transfusion purpura
**(2) Severe TCP (platelet nadir <20,000/mm^3^)**	
Concomitant chemotherapy may cause similar TCP severity	
**(3) Quick rate of decline of platelet counts**	
Decrease of platelet counts over a few days or weeks with no alternative explanation	
**(4) Positive detection of antiplatelet antibodies**	
Most drug-induced immune TCPs are caused by drug-dependent antibodies specific for the drug structure that react to platelets only in the presence of the drug. Antiplatelet antibody testing may help to document the cause of TCP, the causative drug, and which platelet glycoprotein is being recognized [αIIb/ß3 integrin (GPIIb/IIIa), GPIb/IX, etc.,] but is technically demanding, not useful in the immediate clinical decision making, and there may be false negatives even when clinical scenario is highly suggestive of drug-induced immune TCP	
**(5) Bone marrow aspirate and/or biopsy compatible with peripheral (not central) TCP**	
Increased number of megakaryocytes without abnormal cells in bone marrow, with increased percentage of immature platelets in peripheral blood, indicating increased thrombopoiesis	
**(6) Temporal relationship with onset of TCP after ICI administration**	
Most reports describe an early onset within days or weeks of initial cycles although later onset (even months after treatment cessation) has been described. It may occur after initial ICI exposure or after re-exposure following a treatment interruption	
**(7) Recovery of platelets after stopping the ICI**	
Complete and sustained recovery of platelet counts spontaneously after permanent interruption of the ICI, with or without additional steroids or immunosuppressants	
**(8) Effectiveness of corticosteroids and immunosuppressants**	
Quick and effective response to steroids, intravenous immunoglobulins or other immunosuppressors such as cyclosporine or rituximab	
**(9) Ineffective platelet transfusions**	
Failure of platelet transfusions to produce a sustained increase in platelet count because the antiplatelet antibodies cause destruction of the transfused platelets	
**(10) Personal or family history of autoimmune disease**	
Psoriasis, vitiligo, ITP, and Hashimoto thyroiditis have been described in ICI-treated patients diagnosed with ir-TCP	
**(11) Positive autoantibodies in the immunologic screening**	
Antinuclear antibodies, lupus anticoagulant, anti-cardiolipin/antiphospholipid autoantibodies, and others have been detected in the serum of ICI-treated patients who developed ir-TCP. However, positive results are not confirmatory of autoimmune disease (ir-TCP) because autoantibodies may be present in healthy individuals and in patients with non-autoimmune diseases, but provide further evidence	
**(12) Positive direct antiglobulin test (Direct Coombs Test)**	
May indicate simultaneous autoimmune hemolytic anemia (Evans Syndrome) and be supportive of a diagnosis of TCP of immune origin	
**(13) Occurrence of other ir-AEs in the same patient**	
Prior or concomitant neurological, endocrine, gastrointestinal, liver and skin ir-AEs have been described in ICI-treated patients diagnosed with ir-TCP and may raise clinical suspicion of a diagnosis of ir-TCP	

*TCP, thrombocytopenia; ir-TCP, immune-related thrombocytopenia; MDS, myelodysplastic syndrome; MPD, myeloproliferative diseases; TMA, thrombotic microangiopathy; TTP, thrombotic thrombocytopenic purpura; DIC, disseminated intravascular coagulation; HIV, human immunodeficiency virus; HCV, hepatitis C virus; CMV, cytomegalovirus; EBV, Epstein-Barr virus; CLL, chronic lymphocytic leukemia, HD, Hodgkin’s disease; NHL, non-Hodgkin’s lymphoma; LGL, large granular lymphocytosis; SLE, systemic lupus erythematosus; APS, antiphospholipid syndrome; ICI, immune checkpoint inhibitor; HIT, heparin-induced TCP; ITP, immune thrombocytopenia; IVIG, intravenous immunoglobulin; AEs, adverse events; PD1/PD-L1, programmed cell death receptor-1 or ligand-1*.

## Predictive Biomarkers of Immune-Related Thrombocytopenia

No predictive biomarkers can reliably identify patients at risk of ir-TCP. Upregulation of PD-1 expression on B-cells prior to ICI treatment (Kanameishi et al., 2016), the detection of autoantibodies (e.g., antinuclear antibodies, lupus anticoagulant, anti-cardiolipin, antiphospholipid, rheumatoid factor and anti-cyclic citrullinated peptide) (Le Burel et al., 2017), and the presence of specific single nucleotide polymorphisms of PD-1 and CTLA4 possibly associated with certain autoimmune diseases including immune thrombocytopenic purpura (ITP) (e.g., PD-1 +7209 C/T SNP and an increased susceptibility to ITP, CTLA4 -1577 A/G and CT60 A/G associated with ITP severity, and PD-D1 -606 G/A and +63379 C/T associated with the patient’s ITP response to prednisolone) (Kasamatsu et al., 2018), have been postulated as potential biomarkers. However, these findings are not sufficiently conclusive to trigger any practical recommendation and it is unknown whether they can be extrapolated to the risk of ir-TCP.

## Risk Factors for Immune-Related Thrombocytopenia

Various risk factors may increase the susceptibility to develop ir-AEs with ICI therapy. Prior immune-related toxicity with a previous course of immunotherapy (Champiat et al., 2016) and a personal or family history of autoimmune disorders (Bagley et al., 2016; Johnson et al., 2016; Gutzmer et al., 2017; Le Burel et al., 2017; Menzies et al., 2017; Pföhler et al., 2017; Jotatsu et al., 2018) are currently being assessed; if present, the patient’s benefit-risk should be reassessed prior to use of ICIs. Immune-related cytopenias that develop during ICI treatment are common in patients with a prior diagnosis of psoriasis, vitiligo, ITP, or Hashimoto’s thyroiditis, the presence of cold agglutinins, or prior treatment with temozolomide chemotherapy (Ku et al., 2010; Ahmad et al., 2012; du Rusquec et al., 2014), perhaps supporting the link with autoimmune diseases.

Certain concomitant medications that affect the immune system (e.g., steroids, allopurinol, non-steroidal anti-inflammatory drugs, salicylates, metformin, etc.) or that are associated with drug-induced immune TCP (e.g., quinine, quinidine, cotrimoxazole, gold salts, oral antidiabetics, colchicine, etc.) may increase the potential for drug-associated autoimmunity (Solomon, 2015; Champiat et al., 2016). However, these risk factors are not determinant and, thus far, risk mitigation strategies cannot be based solely on excluding them.

## Standard Assessments to Evaluate Suspected Immune-Related Thrombocytopenia

The initial standard work-up when ir-TCP is suspected should include a patient’s detailed medical and family history to help inform on potential risk factors such as history of autoimmunity or certain prior or concomitant medications, a nutritional evaluation and physical examination, and a basic laboratory panel with complete blood count (CBC) and differential, peripheral blood smear, reticulocyte count, direct antiglobulin test, and infection panel. When results are still inconclusive, a bone marrow examination may be helpful to differentiate central versus peripheral TCP. These first-tier evaluations are usually sufficient to establish the most likely diagnosis and guide clinical management decisions. Second-tier assessments, based on initial lab results and clinical suspicion, can provide further evidence to exclude or confirm alternative TCP causes such as heparin-induced TCP (anti-PF4 antibodies), certain infections (additional virus/bacterial panel), disseminated intravascular coagulation (coagulation parameters), thyroid dysfunction (antithyroid antibodies with thyroid function), and importantly, immune TCP induced by concomitant medications (specific drug-dependent antiplatelet antibodies). Table 2 summarizes the recommended evaluations. While standard assessments should be performed as soon as an unexplained drop in platelet count is detected, the timing for complementary assessments is usually guided by the initial lab results and the patient’s clinical evolution.

**Table 2:** Recommended investigations for suspected immune-related thrombocytopenia.

Clinical evaluation	
**Parameter**	**Points to consider**
Patient history	Personal history of autoimmune disease (Hashimoto’s thyroiditis, ITP, vitiligo, psoriasis); prior chemo-radiotherapy or lymphocyte-depleting therapy (fludarabine, anti-thymocyte globulin, corticosteroids)
Family history	History of autoimmune disease
Nutritional evaluation	Iron, folate, vitamin B12 deficiency
Physical examination	Splenomegaly, hepatomegaly, purpura, petechiae

**Standard investigations**	

**Investigation**	**Points to consider**
Complete blood count	Other hematological cell lines decreased (coexistence of neutropenia and/or anemia)
Peripheral blood smear	Platelet clumping (pseudo-TCP), morphology of platelets (myelodysplastic syndrome)
Reticulocyte count	Coexistence of autoimmune hemolytic anemia (Evans syndrome)
Direct antiglobulin test [direct Coombs test]	Coexistence of autoimmune hemolytic anemia (Evans Syndrome)
Infection panel	HIV, HCV, *H. Pylori*
Bone marrow examination (aspirate/biopsy)	Not always necessary; indicated when other investigations are not conclusive or if other cell lines are affected and there is concern for drug-induced aplastic anemia or bone marrow pathology

**Optional investigations**	

**Investigation**	**Points to consider**
Platelet antibody identification panel/drug-dependent antiplatelet antibodies	Human platelet antibodies HPA-1a/b, HPA-2a/b, HPA-3a/b, HPA-4a, HPA-5a/b, GPIIb/IIIa, GPIa/IIa, GP Ib/IX, GPIV, and class I HLA
Anti PF4	Heparin-induced TCP
Additional virus panel	Parvovirus B19, EBV, CMV
Coagulation parameters	PT, aPTT, fibrinogen, D-dimers: rule out TMA/TTP/DIC
Autoantibody screening	Antinuclear antibodies and antiphospholipid autoantibodies (including lupus anticoagulant and anti-cardiolipin)
Antithyroid antibodies and thyroid function	Coexisting autoimmune thyroid disorders (e.g., subclinical hypothyroidism associated with ITP: treating the underlying thyroid disorder may significantly improve platelet count)

*HIV, human immunodeficiency virus; HCV, hepatitis C virus; TCP, thrombocytopenia; HPA, Human platelet antibodies; GP, glycoprotein; HLA, human leukocyte antigen; TMA, thrombotic microangiopathy; TTP, thrombotic thrombocytopenic purpura; DIC, disseminated intravascular coagulation; HIV, human immunodeficiency virus; HCV, hepatitis C virus; HBV, hepatitis B virus; CMV, cytomegalovirus; EBV, Epstein-barr virus; HIT, heparin-induced TCP; ITP, immune thrombocytopenia*.

## Signal Detection and Assessment of Immune-Related Thrombocytopenia in Clinical Development Programs

### Signal Detection

Safety assessment is an integral part of any clinical development program. To identify a safety signal of ir-TCP in ICI clinical programs, it is necessary to first define the platelet threshold that constitutes TCP.

First, 100,000 cells/mm^3^ is the cut-off to define primary immune TCP (Rodeghiero et al., 2009), which could certainly be used for ir-TCP signal detection. This threshold avoids concerns over cell count reductions where platelets remain above 100,000 cells/mm^3^ which usually do not carry any risk for serious bleeding. In line with this platelet cut-off value, the American Society of Clinical Oncology (ASCO) guidelines for the management of ir-AEs in patients treated with ICIs provide specific recommendations for ir-TCP when platelet counts decrease below 100,000 cells/mm^3^. Hence, a reduction in absolute platelet counts below 100,000 cells/mm^3^ seems a reasonable threshold to use for thrombocytopenia signal detection.

Secondly, in the context of immunotherapy, certain platelet count decreases relative to baseline values should also constitute a warning for the physician, even if the platelet count remains within the normal range. This may be particularly useful for signal detection in patients who initiate treatment with a certain degree of TCP as a sequela from prior treatments, for whom a new onset ir-TCP may be partially masked by the pre-existing TCP. In this context, a relative decrease more than 50% over the platelet baseline value may be a useful indicator of a potential newly developing ir-TCP.

Therefore, for optimal TCP signal detection and platelet monitoring during ICI therapy, both an unexplained decrease in absolute platelet counts below 100,000 cells/mm^3^ and/or a reduction of ≥50% from the individual patient’s baseline level are important, and either one should warrant a closer evaluation and more frequent patient monitoring.

#### Search Strategy for Safety Data Retrieval in Clinical Development Programs

For investigation of drug safety concerns and data retrieval and presentation from individual clinical trials and across clinical development programs, it is necessary to identify and aggregate data from all ir-TCP reported cases. A comprehensive search of the safety databases should include a combination of Preferred Terms (PTs) and Standardized MedDRA Queries (SMQs) from the Medical Dictionary for Regulatory Activities (MedDRA). For example, based on the MedDRA browser version at the time of writing this article, the search should include the “haematopoietic cytopenias affecting more than one type of blood cell” (SMQ), the “haematopoietic thrombocytopenia” (SMQ), and two additional PTs: “immune thrombocytopenic purpura” and “thrombocytopenic purpura.” Of note, the search should be revised as new MedDRA versions become available since new terms may be added and others become obsolete.

### Signal Assessment

#### Causality Assessment

Causal attribution of TCP to the ICI during therapy may be confounded by the underlying malignancy (disease progression and bone marrow infiltration) and concomitant medications (drug-related TCP), among others. Table 1 proposes a criteria checklist suggestive of ir-TCP during ICI therapy. Any patient who develops unexplained TCP should be evaluated for these factors and a possible diagnosis of ir-TCP ruled out. When the diagnosis of drug-induced ir-TCP is highly suspected in a pluri-medicated patient, it may be virtually impossible to be identify the causative drug. Investigation of drug-dependent antiplatelets antibodies in serum and platelet-bound antibodies at the time of diagnosis of TCP may help confirm the etiology but requires significant time and is not useful for management decisions.

#### Systematic Evaluation Across Cases: Adverse Event Reporting Versus Laboratory Values

TCP is relatively frequent in oncology clinical trials of ICIs, but it may not always be accurately or consistently reported as an AE when applicable. For this reason, evaluation of changes in laboratory parameters during treatment (e.g., study and program-level shift tables for platelet counts from baseline to the worst and to the last platelet value) may provide more reliable and complementary information on the compound’s potential to cause TCP and its reversibility.

#### Severity Grading of Immune-Related Thrombocytopenia

The CTCAE grading is helpful in guiding dosing decisions in oncology practice and in standardizing the reporting of toxicity across clinical development programs. However, its reliability for evaluating the occurrence and severity of ir-TCP during ICI therapy is questionable as it is based on absolute platelet counts and does not reflect relative changes in platelet counts from baseline. Despite this limitation, there is currently no alternative tool for measuring platelet shifts possibly associated to ICIs.

## Risk Management and Potential Mitigation Strategies

### Screening for Patients “at Risk” and Risk Mitigation

The lack of laboratory or clinical parameters that can predict the risk of developing ir-TCP or its severity prevents implementing reliable de-risking strategies. A certain predisposition to hem-irAEs during PD1/PD-L1 therapy in patients with a personal or family history of autoimmune disease raises the possibility of screening for at-risk patients by testing for certain autoantibodies (Le Burel et al., 2017). An additional de-risking strategy could consist of intensifying the clinical (more frequent patient visits) and biological (more frequent CBCs) monitoring in patients who present:

(a) A positive autoantibodies screening prior to ICI treatment (e.g., antinuclear antibodies, lupus anticoagulant, anti-cardiolipin, antiphospholipid autoantibodies, rheumatoid factor, and anti-cyclic citrullinated peptide).
(b) An increase in absolute eosinophil and lymphocyte count at 1 month after ICI therapy initiation, which have been correlated to an increased risk of all ir-AEs (Schindler et al., 2014; Diehl et al., 2017), possibly based on the immunological functional links between IL-17 and eosinophils, and their correlation with ir-AEs.

However, there is not enough evidence to support a robust association between these biomarkers and risk of ir-TCP, or to reliably use them for patient selection and risk minimization. Therefore, patients should not be denied treatment with ICIs based on positive results because in cancer patients the risk/benefit balance should go toward the cancer treatment.

Finally, a key component of risk mitigation is to ensure that both physician and patient are aware of the possible occurrence of less common yet potentially life-threatening ir-AEs such as ir-TCP. Patients should be educated to recognize clinical signs and symptoms of a possible developing TCP such as mucosal bleeding, petechiae, macroscopic hematuria, buccal hemorrhagic bulla, purpura or any type of abnormal bleeding, and should be informed that most of these events are reversible if detected early and properly treated.

### Patient Monitoring

Patients should be under close monitoring at the beginning, during, and even after ICI treatment discontinuation as ir-TCP may occur at any time and there are no predictive biomarkers of risk. Some experts recommend continued monitoring until 1 year after ICI discontinuation (Champiat et al., 2016).

Clinicians should monitor for signs/symptoms of TCP (easy bruising, petechiae, spontaneous mucocutaneous bleeding) at each visit and advise patients to immediately report abnormal bleeding. A CBC should be performed at baseline and prior to each dose administration, and counts should always be compared with baseline values to detect any clinically relevant shift in platelet counts. A more frequent CBC may be considered when (a) platelets drop below 100,000/mm^3^ or there is a reduction of more than 50% from baseline values (e.g., depending on workup results, consider monitoring once weekly); and (b) the rate of platelet decline is very rapid and there is clinical suspicion that a severe ir-TCP may be developing (e.g., monitor twice weekly or more often if clinically indicated).

Once the possible diagnosis of ir-TCP is established, the patient should be monitored for signs of bleeding (e.g., mucosal bleeding, petechiae, hematuria, buccal hemorrhagic bulla, and very specially “wet purpura” which is associated with a high risk of severe bleeding). CBCs should be performed as clinically indicated until resolution of the TCP or until reaching a stable platelet count, preferably ≥100,000/mm^3^ or baseline values in those patients who had some degree of TCP before treatment initiation.

In terms of event resolution, clinicians should be aware that the strategy and time needed for ir-TCP recovery can highly vary across patients, with platelet counts rising in some patients as early as day 2 with normalization by day 4 (Le Roy et al., 2016), and others necessitating up to 3 months for normalization (Pföhler et al., 2017). Although mild ir-TCP may resolve spontaneously with temporary or permanent treatment discontinuation (Shiuan et al., 2017; Jotatsu et al., 2018), the most comprehensive series characterizing hem-irAEs associated with anti PD-1/PD-L1therapy recently published by Delanoy et al. (2019) described that 78% of ir-TCP cases were grade 4, 100% of patients received steroids, 67% received additional intravenous immunoglobulin (IVIG), and 22% did not respond to steroids or IVIG and required treatment with thrombopoetin agonists or rituximab. Additionally, this study provided preliminary data about the safety of ICI rechallenge in patients with ir-TCP: 67% patients discontinued the ICI permanently and 33% of patients were rechallenged, of which 33% had a recurrence of ir-TCP after the rechallenge. Although the data is too scarce to draw any definite conclusion, the risk of ir-TCP recurrence after rechallenge must be considered, and increased vigilance is warranted. In this series, 78% of patients had resolution of ir-TCP at final follow-up.

## Toxicity Management Guidelines of Immune-Related Thrombocytopenia

Various guidelines are available for the management of ir-AEs including the European Society for Medical Oncology (Haanen et al., 2017), the Society for Immunotherapy of Cancer (Puzanov et al., 2017), and the ASCO (Brahmer et al., 2018) guidelines. On the basis of the ir-TCP severity, the ASCO guidelines recommend early suspension of the ICI when platelets reach <75,000/mm^3^, with initiation of steroids if a quicker platelet count increase is warranted (e.g., abnormal bleeding). Of note, these recommendations are meant for ICIs administered alone and do not provide guidance for ICI combinations with agents with thrombocytopenic liability.

According to the ASCO guidelines, ir-TCP grade 1 (<100,000/mm^3^) should be managed by ICI continuation with close clinical follow-up and laboratory evaluation. Grade 2 (<75,000/mm^3^) requires ICI withholding but monitoring for improvement with interruption until resolution to grade 1, along with administration of oral prednisone with 2–4 weeks tapering and optional IVIG in conjunction with corticosteroids if a more rapid platelet increase is required. For grade 3 (<50,000/mm^3^) and grade 4 (<25,000/mm^3^), a hematology consult is indicated; treatment should be withheld until resolution to grade 1, with administration of high-dose corticosteroids (prednisone 1 to 2 mg/kg/d or methylprednisolone 1 to 2 mg/kg/d) and optional IVIG if a more-rapid platelet increase is required, with permanent discontinuation of ICI there is no improvement. If previous corticosteroids and/or IVIG are unsuccessful, subsequent treatment may include rituximab, splenectomy, thrombopoietin receptor agonists, or more potent immunosuppression.

## Conclusion

Hem-irAEs are potentially life-threatening complications of ICIs that require a high level of clinical suspicion. Ir-TCP is amongst the most frequently reported hem-irAEs. Due to its low incidence and the lack of specific diagnostic criteria, ir-TCP may be underdiagnosed and possibly underreported. The diagnosis is based on exclusion of other causes of TCP together with clinical features that suggest an immune event such as the refractoriness to platelet transfusions and response to corticosteroids. During clinical development, a systematic analysis of ir-TCP cases at clinical study and program level is of outmost importance to better characterize the TCP, understand the risk factors, support causality assessment, and optimize the risk management. In clinical practice, it is critical that both patients and medical personnel involved in ICI therapy be knowledgeable of this rare but potentially serious complication since prompt recognition and initiation of appropriate treatment is key to avoid severe bleeding complications.

## Author Contributions

RC conceived the original idea, reviewed the literature, and wrote and edited the manuscript.

## Conflict of Interest Statement

RC is an employee of AstraZeneca Pharmaceuticals.
